# Structured follow-up by general practitioners after deliberate self-poisoning: a randomised controlled trial

**DOI:** 10.1186/s12888-015-0635-2

**Published:** 2015-10-14

**Authors:** TK Grimholt, D. Jacobsen, OR Haavet, L. Sandvik, T. Jorgensen, AB Norheim, O. Ekeberg

**Affiliations:** Department of Acute Medicine, Oslo University Hospital, Pb. 4950 Nydalen, 0424 Oslo, Norway; Regional Centre of Violence, Traumatic Stress and Suicide Prevention, Eastern Norway, Norway; Department of General Practice, Institute of Health and Society, University of Oslo, Oslo, Norway; Department of Biostatistics Oslo University Hospital, Oslo, Norway; Psychiatric Consultation Team, Akershus University Hospital, Akershus, Norway; Diakonhjemmet Hospital, Oslo, Norway; Department of Behavioural Sciences in Medicine, Institute of Basic Medical Sciences. Faculty of Medicine, University of Oslo, Oslo, Norway

**Keywords:** Adherence, Deliberate self-poisoning, General practitioner, Randomised trial, Satisfaction

## Abstract

**Background:**

General Practitioners (GPs) play an important role in the follow-up of patients after deliberate self-poisoning (DSP). The aim was to examine whether structured follow-up by GPs increased the content of, adherence to, and satisfaction with treatment after discharge from emergency departments.

**Methods:**

This was a multicentre, randomised trial with blinded assignment. Five emergency departments and general practices in the catchment area participated. 202 patients discharged from emergency departments after DSP were assigned. The intervention was structured follow-up by the GP over a 6-month period with a minimum of five consultations, accompanied by written guidelines for the GPs with suggestions for motivating patients to follow treatment, assessing personal problems and suicidal ideation, and availability in the case of suicidal crisis. Outcome measures were data retrieved from the Register for the control and payment of reimbursements to health service providers (KUHR) and by questionnaires mailed to patients and GPs. After 3 and 6 months, the frequency and content of GP contact, and adherence to GP consultations and treatment in general were registered. Satisfaction with general treatment received and with the GP was measured by the EUROPEP scale.

**Results:**

Patients in the intervention group received significantly more consultations than the control group (mean 6.7 vs. 4.5 (*p* = 0.004)). The intervention group was significantly more satisfied with the time their GP took to listen to their personal problems (93.1 % vs. 59.4 % (*p* = 0.002)) and with the fact that the GP included them in medical decisions (87.5 % vs. 54. 8 % (*p* = 0.009)). The intervention group was significantly more satisfied with the treatment in general than the control group (79 % vs. 51 % (*p* = 0.026)).

**Conclusions:**

Guidelines and structured, enhanced follow-up by the GP after the discharge of the DSP patient increased the number of consultations and satisfaction with aftercare in general practice. Consistently with previous research, there is still a need for interventional studies.

**Trial registration:**

ClinicalTrials.gov Identifier: NCT01342809. Registered 18 April 2011.

**Electronic supplementary material:**

The online version of this article (doi:10.1186/s12888-015-0635-2) contains supplementary material, which is available to authorized users.

## Background

Deliberate self-harm (DSH) by poisoning and other methods is a serious health problem worldwide. Repeated DSH, morbidity and mortality are significantly increased [1–3], and repetition rates among patients hospitalised for DSH have been found to be 16 % the first year, with a suicide rate of 7 % over the following nine years [4]. Suicide is one of the leading causes of death in the world, occurring every 40 s [5]. Research is limited and the results are conflicting, and there is therefore insufficient empirical evidence to provide adequate aftercare [6, 7]. Intervention studies involving telephoning patients [8], sending postcards [9, 10], and different forms of psychotherapy have shown reductions in suicidal behaviour [11, 12]. In Norway, no effect was found for assertive outreach, although with a non-randomised design, the results could have been biased [13]. The model was replicated in two Danish trials; the first demonstrated decreased suicidal behaviour [14]. However, when replicated with a randomised design and higher power, the findings were negative [15]. In these trials, it appears that continuity of care, availability in times of crisis, and close contact with the patients at risk were important elements in preventing further suicidal behaviour [12, 10, 14, 16]. The role of the general practitioner (GP), an already established and available health care service in many countries, is consistent with these elements. Further, the establishment of aftercare for patients hospitalised for DSP is important, because many receive no psychiatric follow-up, and report excessive waiting time and the need for improved access to aftercare [17]. Adherence to the planned treatment is also a problem [18]. The high proportion of suicidal patients who make contact with GPs both before and after an episode of DSH [17, 19, 20] and their diverse needs, underline the fact that more research is needed on how best to manage such patients in primary care. GPs can represent a stable long-term contact and ensure a holistic approach by coordinating and implementing additional services. In spite of this, there have been a limited number of clinical trials aiming to study follow-up in primary care and the potential of the GPs.

In a randomised controlled trial (RCT), the GPs received guidelines and an invitation letter to be sent to DSH patients after discharge from hospital [21]. This had no effect on reducing repeated self-harm. However, the question of whether the intervention would have had an effect on outcome variables based on patients’ perceptions remains. Patients’ evaluations of the treatment they receive from different health care services are relevant to the development of high quality care. However, in a study performed more than 25 years ago, outpatient counselling was compared to aftercare in general practice. Here, there were no significant differences between the groups regarding whether they had found their treatment from GP or out patient clinic helpful, or whether suicidal behaviour decreased [22].

Based on the current status, a RCT was designed. The intervention comprised a scheduled appointment with a GP within 1 week of discharge and at least five scheduled consultations over the next 6 months. The GPs received guidelines with suggestions for assessing and managing patients. Our aim was to examine whether this structured intervention will be associated with a greater increase in patient satisfaction and adherence to treatment provided by a GP than treatment as usual.

### Methods

A controlled clinical trial was performed at five hospitals in Oslo and Akershus County, Norway. Oslo University Hospital Ullevaal, Aker University Hospital, Diakonhjemmet Hospital, Lovisenberg Hospital and Akershus University Hospital.

Patients were randomised to intervention or treatment as usual. Patients whose physicians refused to participate were studied as a comparison group. The manuscript reporting adheres to the CONSORT guidelines.

### Inclusion criteria

DSP was defined in accordance with the WHO/EURO Multicentre Study on Suicidal Behaviour [23], but included only patients with poisoning.

The inclusion criteria were: aged 18–75, registered with a GP, and discharged directly to home, thus enabling follow-up by a GP.

### Exclusion criteria

Excluded were patients with present psychosis, mental retardation, organic cognitive impairment and those unable to fill in the questionnaire because it was not written in their native language.

### Recruitment and participants

Prior to the trial, information and an invitation letter were sent to all the registered GPs in the hospitals’ catchment areas. The patients were recruited from medical departments after an episode of DSP. When a patient was ready for an interview in the hospital, usually after the psychiatric assessment, he or she was informed about the trial and invited to participate. The project coordinator telephoned the GPs as soon as a patient was assigned to intervention and explained the rationale of the trial. Those who agreed to participate were sent information, guidelines, and a registration form. The GPs in the control group were mailed a registration form and a letter describing the trial in general and were asked to treat the patients as usual.

Excluded were the GPs that either refused to participate beforehand or when approached by telephone and asked about participation. Their patients were followed in a comparison group.

### Randomisation

Sealed envelopes with randomisation codes generated from randomization.com were used. The codes were stored separately at the hospital department secretary’s office to ensure that no manipulation occurred after the assignment to groups.

### Blinding

The assignment personnel and the patients were subjected to blinding at the time of inclusion in accordance with Zelen randomisation [24] to ensure the concealment of allocation and thus control for selection bias. Because of the nature of the study, the patients were not subjected to blinding during the study period.

### Intervention

The intervention consisted of systematic follow-up for 6 months after discharge. The GP was instructed to contact the patient and schedule an appointment as soon as possible, preferably within 1 week of discharge. Further, a minimum of five consultations during the 6-month period after discharge was recommended. GPs received written guidelines with suggestions of topics to discuss with the patient during the consultation. In addition, and where possible, a discharge summary from the medical department was sent to the GP immediately after discharge. See Additional file 1.

### Treatment as usual

The control and comparison groups received treatment as usual. Arrangements of referrals to appropriate health care services were based on the assessment by the psychiatric liaison teams and the discharging physician in the hospital.

### Measures

At baseline in the hospital, the patients filled out a questionnaire with demographic background information on their marital, living, educational and employment status, and on previous DSH and psychiatric treatment. Questions from the European Parasuicide Study Interview [25] were used to describe treatment in general practice before and after the hospitalisation, and whether the patients communicated suicide ideation.

Three and six months after discharge from hospital, the patients were mailed a questionnaire and at the same time telephoned by the study team. Adherence to the scheduled appointments with the GP was measured with the question “Have you scheduled an appointment with your GP but not kept it?” The number of dropouts was measured with the question “To what extent have you met your scheduled treatment appointments?”

Satisfaction with the GP was measured with the Norwegian version of the European Task Force on Patient Evaluations of General Practice (EUROPEP) [26]. The questionnaire consists of a set of 25 items using a five-point scale. Extremes are labelled as ‘1 = poor’ and ‘5 = excellent’ on evaluations of different aspects of care: Communication, the GP listening to patients, time spent in consultations, and speed of response in cases of urgent problems. See Additional file 2. Satisfaction with follow-up in general was measured by the question “Overall, how satisfied are you with the follow-up you have received?” measured on a four-point Likert scale from 1 = very satisfied to 4 = very dissatisfied.

Additional information about health care services received was also added to the questionnaire.

We sent registration forms to 171 GPs in the intervention and control groups when their patients were assigned to the trial. The overall response rate was 47 % (*n* = 87). There were 44 GPs in the intervention, and 43 GPs in the control group who returned completed forms.

The variables in the registration form to the GPs were: the number of consultations, telephone contact, whether the patient had dropped out of appointments and been provided with a rescheduled appointment, and whether the GP had contacted the study team or others to discuss challenges related to the patient.

In addition, the intervention GPs reported the time from hospital discharge until the first consultation and the content of the consultations during the study period.

### Registry data

We retrieved data on all contact between the patients and their GP during the 6-month period after discharge from The Norwegian Register for the control and payment of reimbursements to health service providers (KUHR). This register receives all refund applications from the GPs. The data were recoded and categorised into: Simple contact (without attending the GPs office), normal consultation, extended consultation, consultation with therapy or use of psychometric assessment tools or other tasks related to psychiatric diagnoses, out-of-office consultation, extended out-of-office consultation (mainly somatic outpatient visits or home visits), talking to relatives, filling out forms, and other contacts (sampling, procedures, etc.).

The refund applications were also provided by ICPC-2 diagnoses related to each activity.

P (psychiatric), Z (social problems) and A-86 (poisoning) diagnoses were combined into the category psychosomatic, and all other diagnoses were categorised as somatic.

### Statistical power

Suicidal ideation was considered as the primary outcome measure (this will be addressed in another paper). A difference of 5 points on the Beck Scale for Suicidal Ideation was considered to be clinically significant. Assuming α = 0.05 and β = 0.2, and allowing for a one-third dropout rate, the calculated sample size was 60 patients to each group.

### Statistics

Descriptive statistics, means and frequencies were used. Chi-square and Fisher’s exact tests were used to compare the categorical data. EUROPEP data were dichotomised into item 1–3 (dissatisfied–neutral) and 4–5 (satisfied), and chi-square tests used to compare all the items. The do not applicable scores were replaced with missing. The non-applicable scores were treated as missing data. The comparisons of continuous variables were conducted by means of independent sample t-tests. All statistical tests were two-sided and p-values less than 0.05 were considered statistically significant. SPSS v.21 (Chicago, IL) was used for statistical analyses.

### Ethics

The Regional Ethics Committee, Region of Eastern Norway, approved the study protocol for all the five centres. Further the Personal Privacy Protection Agency at Oslo University Hospital approved the study. The patients were informed verbally and signed a detailed written consent in the hospital after recovery from the acute poisoning. It was important to verify that registered patients were still alive before mailing them a questionnaire. The participants were telephoned by a health care professional in the study team at the time they received the questionnaire and asked whether they had questions related to it, or if they wished to provide their answers by telephone. If the patients were severely depressed or considered to have an ongoing suicidal crisis, health care assistance was provided. During the study period, the patients were given a telephone number to contact the project leader if they had any questions or wished to leave the trial.

We were not allowed to register personal security :numbers to gather information about the patients who did not meet the inclusion criteria or declined to participate. The Ethics Committee did not approve the proposed use of a twofold written consent incorporating a request for those who declined to participate in the trial to complete a baseline questionnaire at the hospital.

## Results

### Sample and baseline characteristics

**Fig. 1:**
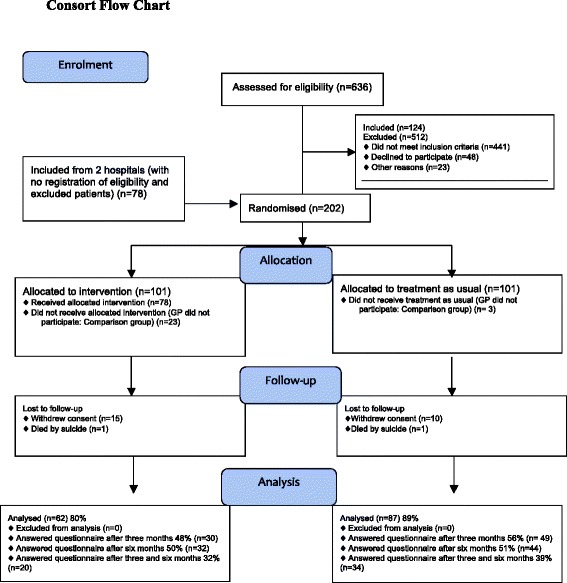
Consort flow chart

Among the 202 included patients, 101 were randomised to intervention and 101 to the control group. Twenty-six were excluded due to non-participation of their GP (Fig. 1).

There were no significant differences between the intervention group and the control group in terms of baseline characteristics (Table 1). The majority were female. About 50 % had previously been hospitalised for DSH. More than 50 % had received treatment in psychiatric care.

**Table 1:** Demographic and clinical characteristics

	Intervention group (*n* = 62) % (*n*)	Control group (*n* = 87) % (*n*)	*P* value
Gender			
Female	77.4 (48)	72.4 (63)	
Male	22.6 (14)	27.6 (24)	0.490
Mean age (95 % CI)	35.6 (32–39)	40 (36–40)	0.081
Marital status			
Single	50.0 (30)	40.5 (34)	
Married/cohabiting	43.4 (26)	41.7 (35)	
Separated/divorced	6.7 (4)	13.1 (11)	
Widowed	0.0 (0)	3.6 (3)	0.312
Living status			
Alone	37.7 (23)	39.7 (29)	
With others	62.3 (35)	60.3 (48)	0.959
Education			
Elementary high school	12.9 (8)	25.9 (22)	
Secondary vocational	54.8 (34)	49.4 (42)	
College/university	32.3 (20)	24.7 (21)	0.143
Employment status			
Employed, student, military	49.2 (30)	40.5 (34)	
Unemployed	14.8 (9)	13.1 (11)	
Sick leave	6.6 (4)	11.9 (10)	
Welfare recipient	23.0 (14)	13.1 (11)	
Retired	0.0 (0)	7.1 (6)	
Maternity leave	1.6 (1)	1.2 (1)	
Other	4.9 (3)	13.1 (11)	0.103
Previously hospitalised for DSH			
No	38.2 (21)	48.1 (37)	
Yes	61.8 (34)	51.9 (40)	0.260
Previous psychiatric outpatient treatment			
No	44.3 (27)	45.1 (37)	
Once	11.5 (7)	17.1 (14)	
2–3 times	14.8 (9)	9.8 (8)	
4 times or more	29.5 (18)	28.0 (23)	0.679
Previous psychiatric inpatient treatment			
No	59.0 (36)	69.9 (58)	
Once	9.8 (6)	12.0 (10)	
2–3 times	14.8 (9)	9.6 (8)	
4 times or more	16.4 (10)	8.4 (7)	0.326
Want help to solve the problems that triggered the current episode			
No	9.8 (6)	6.2 (5)	
Yes	90.2 (55)	93.8 (76)	0.419

Dropout analyses showed no significant differences in baseline characteristic variables between the participants and the patients that withdrew their consent during follow-up. There were no significant differences in baseline characteristics between the participants and those not included.

### Reason for consultation and communication of DSH thoughts to GP before index episode

The vast majority of patients had consulted their GP in the last year, and one-third of them had done so less than 1 week before the episode (Table 2). There were no differences between the study groups at baseline.

**Table 2:** Consultations and communication of DSH thoughts to GP

	Intervention group % (*n*)	Control group % (*n*)	*P* value
GP consultations in last year before DSH episode			
No consultations	11.5 (7)	13.3 (11)	
1–5 consultations	54.1 (33)	50.6 (42)	
> 5 consultations	34.4 (21)	36.1 (30)	0.905
Last consultation before DSH episode			
< one week	31.7 (19)	24.7 (19)	
1–4 weeks	38.3 (23)	45.5 (35)	
> 4 weeks	30.0 (18)	29.9 (23)	0.607
Reason for last consultation			
Somatic	41.7 (25)	46.9 (38)	
Psychiatric	21.7 (13)	23.5 (19)	
Both	36.7 (22)	29.6 (24)	0.676
DSH thoughts at last consultation			
No	74.2 (46)	70.4 (57)	
Yes	25.8 (16)	29.6 (24)	0.614
Talked with GP about DSH thoughts in last consultation before DSH episode			
No	50 (8)	42.9 (9)	
Yes	50 (8)	57.1 (12)^a^	
Talked with GP but did not report thoughts	1/46	2/57	

^a^Missing *n* = 3

One-quarter of the patients had DSH thoughts during the last consultation; and 50 % in the intervention group and 57.1 % in the control group discussed their DSH thoughts with their GP during the last consultation before the episode. The proportion of patients in both groups that communicated suicidal thoughts to the GP in the last month before the overdose in the current trial was 54.8 % (35.5 % implied and 19.4 % told the GP directly).

### Thoughts of DSH and communication during the six months follow up period

During follow-up, 40.9 % in the intervention group and 47.8 % in the control group had thoughts of DSH and talked with their GP about them (*p* = 0.641). Of those who had thoughts about DSH, 13 out of 31 (41.7 %) discussed them with the GP. In addition, 5 out of 12 (41.7 %) discussed thoughts about DSH even if they did not report such feelings.

### Contact with GP during the six-month post-discharge period

Registry data from KUHR (Table 3) includes information about contact and other activities performed by the GPs during the six-month follow-up period. There was significantly higher frequency of therapy and use of psychometric assessment tools in the intervention group (mean 3.2 vs. 1.8, *p* = 0.037). The total number of consultations was significantly higher in the intervention group (mean 6.7 vs. 4.5, *p* = 0.005). There was also a higher number of activities registered with the ICPC-2 psychiatric, social and poisoning diagnoses (mean 8.6 vs. 5.5 *p* = 0.007), as shown in Table 4. During this period, 64 % in the intervention group and 52 % in the control group had telephone contact with their GP (*p* = 0.276). The mean time from discharge to first consultation in the intervention group was 7 days. (Data were not provided from the GPs in the control group.)

**Table 3:** Comparison of ICPC-2 diagnoses and contact with GP during six-month post-discharge period

	Intervention *N* = 62 Mean (SD)	Control *N* = 87 Mean (SD)	*p* value
ICPC-2 diagnoses reported from all GP contact			
Somatic	4.0 (3.9)	3.6 (4.1)	0.605
Psychiatric, social, or poisoning	8.6 (7.3)	5.5 (6.5)	0.007
Contact with GP			
Consultations^a^	6.7 (5.0)	4.5 (4.2)	0.005
All other patient-related activities^b^	5.9 (6.1)	4.6 (5.4)	0.174

^a^ Normal consultations and extra fees applied because of extended time spent in consultations (over 20 min), therapy, and use of psychometric assessment tools or other consultations related to psychiatric diagnoses

^b^ Simple contact without attending GPs office, outside office consultations; home visit or consultation in somatic outpatient clinic, talking with relatives, contact with other services and all other patient-related activities

**Table 4:** Self-reported treatment as ususal received post-discharge

	Intervention group % (*n*)	Control group % (*n*)	*p* value
3 months post-discharge			
Conversational therapy	76.7 (23)	77.8 (35)	0.910
Pharmacotherapy	53.3 (16)	51.1 (23)	0.850
Psychiatric outpatient clinic	48.4 (15)	57.1 (28)	0.444
3 to 6 months post-discharge			
Conversational therapy	71.4 (20)	58.8 (20)	0.302
Pharmacotherapy	57.1 (16)	38.2 (13)	0.138
Psychiatric outpatient clinic	38.7 (12)	45.2 (19)	0.577

### Treatment as ususal

Apart from the significantly higher number of consultations with GPs in the intervention group, the treatment received from different health care services did not differ between the two groups. GP visits were most frequent, followed by psychiatric outpatient clinic visits. Table 4 displays the prevalence of received conversational therapy, pharmacotherapy and treatment in psychiatric outpatient clinics. There were no significant differences between the study groups after 3 and 6 months.

There were no significant differences between the study groups in patients reporting having received treatment with psychotherapy, abuse related treatment, and economical or family counselling.

During the trial, two GPs contacted the study team to discuss problems related to a patient and one discussed problems with another colleague.

### Adherence

The dropout rate from the scheduled consultation reported by the GPs was 34 % in the intervention group and 44 % in the control group (*p* = 0.593).

Eighty-six percent of the intervention group received a new rescheduled appointment after dropout compared with 67 % of the control group (*p* = 0.365).

The prevalence of patients reporting after 3 months that they had not dropped out from planned treatment was 83 % in the intervention group and 73 % in the control group (*p* = 0.086). After 6 months, these numbers were 76 % and 73 %, respectively (*p* = 0.560).

### Satisfaction

**Fig. 2:**
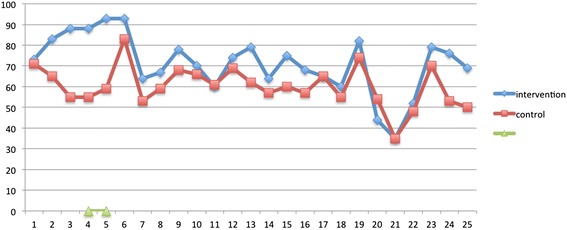
Satisfaction with general practitioner

The scores on the EUROPEP scale after 6 months were high in both groups, indicating a high degree of satisfaction with all aspects of general practice (Fig. 2).

As shown in Fig. 2, the items 2–6 in the EUROPEP scale related to the presence of a good doctor–patient relation, with the items regarding the GP having interest, making it easy to talk, and listening to problems being higher in the intervention group than in the control group. Those in the intervention group were significantly more satisfied with the GP listening to personal problems (93.1 % vs. 59.4 %, *p* = 0.002) and more of them considered that the GP included them in medical decisions (87.5 % vs. 54. 8 %, *p* = 0.009).

The satisfaction with treatment in general was also high in both groups. After 3 months, the prevalence of patients who were somewhat or highly satisfied was 83 % in the intervention group and 68 % in the control group (*p* = 0.158). After 6 months, this difference was significant (79 % vs. 51 % *p* = 0.026).

## Discussion

### Main findings

The main finding of this RCT of DSH patients after discharge was that the prevalence of contact with the GP related to psychosocial problems was significantly higher in the intervention group. The patients were significantly more satisfied with the treatment in general and reported that the GPs listened to their personal problems and involved them in decisions. The findings may contribute to improved and more sustainable aftercare of DSH patients.

Two important aspects related to suicidal behaviour in general practice are availability in time of crisis, and referral to and follow-up of treatment from other health care services, especially related to psychosocial problems. To achieve this, it is important that the GPs devote sufficient time and are interested in the patients’ problems. The findings in this study indicate that these premises were achieved.

The high degree of satisfaction with the care received in general practice in both groups is important and underlines the role of the GP in aftercare of DSH patients, especially given the prevalence of patients that are referred to aftercare with their GP [20, 25].

This is supported by findings that most self-poisoning patients considered their GP to be the most important contact post-discharge [17]. Further, findings from an English study indicated that DSH patients found it helpful and were satisfied when they discussed emotional problems and factors that contributed to the act during follow-up consultations with their GP [25].

Among those who had thoughts about DSH, 54.1 % discussed them with the GP. In addition, 13.6 % discussed thoughts about DSH even if they did not report experiencing such feelings. We do not know the reason for this, but one possibility is that the GP raised the question, perhaps because the patient seemed to be depressed without having active DSH thoughts. These data indicate that the GPs were aware of and motivated to address suicidal issues before the study intervention. Even so, it turned out to be possible for GPs to offer more regular follow-up and address psychosocial issues to a greater extent, which was also associated with more satisfaction among the patients.

The prevalence of discussing suicidal ideation during follow-up would ideally have been higher, especially in the intervention group. It is possible, however, that the climate for discussing such thoughts is strengthened if the patients perceive that the GP has sufficient time and shows an interest in psychosocial problems. Further, a consistent and personal relationship with the GP as the gatekeeper facilitates the communication of suicidal ideation [27]. Further research should explore why patients in the intervention group did not communicate suicidal thoughts to the GP, despite indicating that they were more satisfied with the way the GP listened to their personal problems.

### Strengths and weaknesses

To our knowledge, this is the first time that the impact of structured aftercare in general practice has been examined among DSH patients with satisfaction as one of the outcomes. Further, the KUHR registry data increased the reliability of the number of contacts made with GPs and the specification of whether the contacts were related to psychosocial or somatic diagnoses. The use of registry data also reduced answering bias, because the response rate was lower than expected. These data also enabled us to describe the extent and length of the contact with GPs, which to our knowledge has not been included in previous trials.

Another strength of this trial was the data provided by several health care services on the type and place of treatment for patients, thus enabling us to compare and more clearly differentiate treatment given in the intervention and the control groups. These comparisons have not often been provided in similar RCT studies, but have been requested in reviews [7, 28].

The trial was performed in wards in Oslo and Akershus County, which are specialised in psychosocial assessment and the referral of patients to appropriate treatment after self-poisoning. The suicide motive was assessed, which might have strengthened the inclusion criteria in line with the definition of DSP.

The blinding at the time of assignment prevented bias with regard to the usual treatment recommended in follow-up plans. However the GPs were not blinded, and therefore the comparison group with GPs that did not wanted to participate emerged.

The use of the validated EUROPEP scale also strengthened the reliability of the measured satisfaction with GPs.

### Limitations

There are several limitations to the current study. First, similar to Bennewith’s trial [21], we did not obtain information from the GPs about whether they had actually used the guideline. The patients’ retrospective self-report containing information regarding their treatment and especially the communication of suicidal thoughts should also have been verified by the form sent to the GPs. Further studies should gather more detailed information about how the intervention was carried out.

Nevertheless, the finding that a considerable number of patients did not talk about their DSH thoughts indicates that the guideline was not used consistently. On the other hand, the GPs were encouraged to raise the question of suicidal ideation, to invite the patients to follow-up more actively and to address their compliance with other treatment. This may have helped the GPs to structure the consultations with these patients.

Second, the dropouts and self-reporting response rates during follow-up biased the validity, although there were no significant differences with regard to background variables at baseline. However, the socio-demographic profile was similar to previous studies [14, 29] and strengthens the external validity of the study.

The validity is also to some extent limited by the participation bias from the patients who declined to participate. Although their number was lower than in similar studies, the characteristics of the non-participants should preferably be described in future studies. However, this is difficult in Norway because usually, Regional Ethics Committees do not approve such studies. We did not obtain approval from the Ethics Committee to describe demographic and clinical factors in patients that did not consent to participate in the trial. In a Norwegian study of the same population, although with a different design, the researchers were allowed to obtain information about those who did not participate. They found no significant difference according to gender, but a higher age in the non-participation group [30].

Third, the findings cannot be generalised as applying to patients with higher risk of further suicidal behaviour, such as patients admitted to psychiatric inpatient care [29], those with no fixed abode [30], and DSH patients using violent methods [31–33].

Fourth, the number of participants in the two groups might have been more equal if we had adjusted for the non-participating GPs, although this was difficult to estimate beforehand.

Fifth, the GPs in the control group were not blinded. This might have contributed to performing bias as they could follow their patients more frequent than ususal. As such, the results from the comparison group would therefore be more valid, because the GPs that declined to participate in the pre-trial invitation were not informed again when one of the patient on their list later was included in the trial. However, the comparison group also consisted of GPs that were telephoned during the trial when a patient on their list was randomised into intervention and then declined. Further it can be questioned whether it is ethically correct to blind the participating GPs in a RCT, and therefore all the GPs in the current trial were given information and an opportunity to consent.

Finally, factors that might influence the outcome are not described. Attitudes and personal factors among the GPs that may affect the doctor–patient relationship, how the various problems are addressed, and whether the patients feel that the GP endeavours to understand the individual’s unique history and life circumstances, are all important. Sensitivity to such factors and the ability to handle interpersonally challenging encounters with patients may influence the outcome [34]. However, a comparison of satisfaction between the patients whose GP declined participation and the control group yielded no significant differences in satisfaction or prevalence of contact with the GP during the follow-up. Because all the GPs in the intervention group agreed to participate, it is difficult to specify the number of GPs in the control group who declined because we only knew the number of GPs who declined beforehand by responding negatively to the pre-trial invitation. Given the premise that the responding GPs were more inclined and dedicated to the treatment of suicidal patients, an additional analysis of the GPs in both groups who returned the form after 3 months was performed. No significant differences between the control and the intervention group were found in the outcome variables. However, the rather low sample size in outcome variables from self-reporting increases the risk of type 2 errors, whereas the outcomes from the registry data did not show any differences.

### Findings in relation to other studies

The finding of higher satisfaction in the intervention group is consistent with results from trials in which patients received interpersonal therapy [12] and experimental social work services [35]. In another RCT, where Hawton and colleagues compared outpatient counselling with aftercare in general practice, there were no significant differences in repeated suicidal behaviour or with regard to whether the patients perceived the treatment as helpful [22].

Assertive outreach for poorly compliant patients may be a necessary component in maximising the delivery of any effective treatment [6]. In the present guidelines, suggestions were made for GPs to encourage the patient to follow planned treatment, although the dropout rates did not differ between the intervention and control groups. These findings are in contrast to previous trials with community follow-up of patients who did not attend outpatient appointments [18], and home treatment where substantially increased rates of treatment take-up were found [36].

Availability and assertive outreach after an episode of DSH have shown promising results in decreasing suicidal behaviour [37]. Diverging results were found in two trials of assertive outreach in Denmark; one quasi-experimental study showed a significant reduction in repeated suicidal acts [14], while the replicated randomised trial did not support these results [15]. These trials were based on the Baerum model from Norway, where suicide prevention teams followed patients after discharge from hospitals. However, no significant decline in repeated suicide attempts was found [13].

Bennewith and colleagues were moreover unable to demonstrate any reduction in repeated DSH in one British RCT in general practice, in which they sent a guideline to the GPs [21]. This may indicate that even though it is possible to improve follow-up from GPs, interventions that are more extensive may be necessary to reduce DSH. Results from the current trial where outcome measures are of suicidal symptoms and behaviour will be reported in another paper.

### Clinical implications

The findings related to increased contact with GPs are consistent with one RCT [16] in which the researchers pointed out that their intervention was useful for patients who had never received psychiatric care before their index suicide attempt, and before they were offered contact with health professionals.

Patients that are not already in a treatment programme can be followed by their GP until they receive other scheduled treatment. Improvement is needed, as many patients do not receive follow-up [38, 39]. Active outreach is also important, because almost a third of patients did not report follow-up despite arrangements registered at the hospital [17]. More coordinated and frequent follow-up by the GP could probably reduce this gap in the chain of care. It is important that the hospital aims for a medical review to be sent as soon as possible after discharge, which has been an area in need of improvement [40].

In a study of self-poisoned patients, the waiting time for a follow-up appointment, for example at psychiatric outpatient units, was up to 3 weeks [17]. This is too long for patients in crisis and can be compensated for by consultations with the GP.

The average time from discharge to the first consultation in the intervention group in the current trial was 7 days. This is an advantage when accounting for the elevated risk of suicidal behaviour the first week after discharge [41].

There was a significant higher frequency of ICPC-2 diagnoses of psychiatric, social, or poisoning in the intervention group. The reason for this is not known. One possible explanation could be that these diagnoses were coupled to the planned consultations as part of the intervention. Another possible cause may be be, that the GPs in the intervention group were more aware and thoroughly in their assessments of psychosocial problems among their patients.

### Future research

The need for clinical trials in suicide prevention is highlighted by the WHO’s Mental Health Action Plan 2013–20, where the Member States have committed to work towards reducing the rate of suicide in countries by 10 % by 2020 [5].

The need to target secondary prevention measures in the large group of hospitalised DSH patients should be emphasised and accompanied by financial support for performing and facilitating sufficiently powered multicentre and international cooperation clinical research whereby suicidal behaviour constitutes a primary outcome measure in line with the trial by Fleichmann et al. [42].

The need to provide sufficient health care services for this patient group frequently attending general practice is clear, but more research is neccessary to give firm recommendations. The multifaceted problems and high levels of psychiatric and physical morbidity, social deprivation and suicidal symptoms must be addressed, particularly to prevent the burden on the patients, their partners, children and other relatives. The reasons behind DSH are diverse, and include crisis reactions, severe depression or psychosis; tailored interventions for subgroups may make it easier to show the effect of intervention.

## Conclusion

Structured follow-up by GPs after an episode of DSP was associated with patients having more contact with their GP, and this increase was related to their psychosocial problems. The patients were also more satisfied with their general treatment and the way that the GPs listened to their personal problems and involved them in the decision making.

## Additional files

Additional file 1:
**Guideline intervention.** (PDF 79 kb) Additional file 2:
**Europep questionnaire.** (PDF 38 kb)

## Abbreviations

DSH: Deliberate Self Harm
DSP: Deliberate Self Poisoning
EUROPEP: European Task Force on Patient Evaluations of General Practice
GP: General Practitioner
KUHR: Register for the control and payment of reimbursements to health service providers

## References

[1] Carroll R, Metcalfe C, Gunnell D (2014). Hospital presenting self-harm and risk of fatal and non-fatal repetition: systematic review and meta-analysis. PLoS One.

[2] Bjornaas MA, Jacobsen D, Haldorsen T, Ekeberg O (2009). Mortality and causes of death after hospital-treated self-poisoning in Oslo: a 20-year follow-up. Clin Toxicol (Phila).

[3] Heyerdahl F, Bjornaas MA, Dahl R, Hovda KE, Nore AK, Ekeberg O (2009). Repetition of acute poisoning in Oslo: 1-year prospective study. Br J Psychiatry.

[4] Owens D, Horrocks J, House A (2002). Fatal and non-fatal repetition of self-harm. Systematic review. Br J Psychiatry.

[5] WHO (2014). Preventing suicide: a global imperative.

[6] Hawton K, Arensman E, Townsend E, Bremner S, Feldman E, Goldney R (1998). Deliberate self harm: systematic review of efficacy of psychosocial and pharmacological treatments in preventing repetition. BMJ.

[7] Hawton K, Townsend E, Arensman E, Gunnell D, Hazell P, House A (2000). Psychosocial versus pharmacological treatments for deliberate self harm. Cochrane Database Syst Rev.

[8] De LD, Dello BM, Dwyer J (2002). Suicide among the elderly: the long-term impact of a telephone support and assessment intervention in northern Italy. Br J Psychiatry.

[9] Motto JA, Bostrom AG (2001). A randomized controlled trial of postcrisis suicide prevention. Psychiatr Serv.

[10] Carter GL, Clover K, Whyte IM, Dawson AH, D’Este C (2013). Postcards from the EDge: 5-year outcomes of a randomised controlled trial for hospital-treated self-poisoning. Br J Psychiatry.

[11] Brown GK, Ten HT, Henriques GR, Xie SX, Hollander JE, Beck AT (2005). Cognitive therapy for the prevention of suicide attempts: a randomized controlled trial. JAMA.

[12] Guthrie E, Kapur N, Mackway-Jones K, Chew-Graham C, Moorey J, Mendel E (2001). Randomised controlled trial of brief psychological intervention after deliberate self poisoning. BMJ.

[13] Johannessen HA, Dieserud G, De LD, Claussen B, Zahl PH (2011). Chain of care for patients who have attempted suicide: a follow-up study from Baerum, Norway. BMC Public Health.

[14] Hvid M, Vangborg K, Sorensen HJ, Nielsen IK, Stenborg JM, Wang AG (2011). Preventing repetition of attempted suicide–II,The Amager project, a randomized controlled trial. Nord J Psychiatry.

[15] Morthorst B, Krogh J, Erlangsen A, Alberdi F, Nordentoft M (2012). Effect of assertive outreach after suicide attempt in the AID (assertive intervention for deliberate self harm) trial: randomised controlled trial. BMJ.

[16] Cedereke M, Monti K, Ojehagen A (2002). Telephone contact with patients in the year after a suicide attempt: does it affect treatment attendance and outcome? A randomised controlled study. Eur Psychiatry.

[17] Grimholt TK, Bjornaas MA, Jacobsen D, Dieserud G, Ekeberg O (2012). Treatment received, satisfaction with health care services, and psychiatric symptoms 3 months after hospitalization for self-poisoning. Ann Gen Psychiatry.

[18] Wittouck C, De Munck S, Portzky G, Van Rijsselberghe L, Van Autreve S, van Heeringen K (2010). A comparative follow-up study of aftercare and compliance of suicide attempters following standardized psychosocial assessment. Arch Suicide Res.

[19] Kapur N, House A, Creed F, Feldman E, Friedman T, Guthrie E (1998). Management of deliberate self poisoning in adults in four teaching hospitals: descriptive study. BMJ.

[20] Gunnell D, Bennewith O, Peters TJ, Stocks N, Sharp DJ (2002). Do patients who self-harm consult their general practitioner soon after hospital discharge? A cohort study. Soc Psychiatry Psychiatr Epidemiol.

[21] Bennewith O, Stocks N, Gunnell D, Peters TJ, Evans MO, Sharp DJ (2002). General practice based intervention to prevent repeat episodes of deliberate self harm: cluster randomised controlled trial. BMJ.

[22] Hawton K, McKeown S, Day A, Martin P, O’Connor M, Yule J (1987). Evaluation of out-patient counselling compared with general practitioner care following overdoses. Psychol Med.

[23] Platt S, Bille-Brahe U, Kerkhof A, Schmidtke A, Bjerke T, Crepet P (1992). Parasuicide in Europe: the WHO/EURO multicentre study on parasuicide. I. Introduction and preliminary analysis for 1989. Acta Psychiatr Scand.

[24] Zelen M (1979). A new design for randomized clinical trials. N Engl J Med.

[25] Houston K, Haw C, Townsend E, Hawton K (2003). General practitioner contacts with patients before and after deliberate self harm. Br J Gen Pract.

[26] Grol R, Wensing M, Mainz J, Jung HP, Ferreira P, Hearnshaw H (2000). Patients in Europe evaluate general practice care: an international comparison. Br J Gen Pract.

[27] Osvath P, Michel K, Fekete S (2003). Contacts of suicide attempters with healthcare services in Pecs and Bern in the WHO/EURO Multicentre Study on Parasuicide. Int J Psychiatry Clin Pract.

[28] Arensman E, Townsend E, Hawton K, Bremner S, Feldman E, Goldney R (2001). Psychosocial and pharmacological treatment of patients following deliberate self-harm: the methodological issues involved in evaluating effectiveness. Suicide Life Threat Behav.

[29] Hepp U, Wittmann L, Schnyder U, Michel K (2004). Psychological and psychosocial interventions after attempted suicide: an overview of treatment studies. Crisis.

[30] Gjelsvik B, Heyerdahl F, Lunn D, Hawton K (2014). Change in access to prescribed medication following an episode of deliberate self-poisoning: a multilevel approach. PLoS One.

[31] Mortensen PB, Agerbo E, Erikson T, Qin P, Westergaard-Nielsen N (2000). Psychiatric illness and risk factors for suicide in Denmark. Lancet.

[32] Haw C, Hawton K, Casey D (2006). Deliberate self-harm patients of no fixed abode: a study of characteristics and subsequent deaths in patients presenting to a general hospital. Soc Psychiatry Psychiatr Epidemiol.

[33] Runeson B, Tidemalm D, Dahlin M, Lichtenstein P, Langstrom N (2010). Method of attempted suicide as predictor of subsequent successful suicide: national long term cohort study. BMJ.

[34] Anderson T, Ogles BM, Patterson CL, Lambert MJ, Vermeersch DA (2009). Therapist effects: facilitative interpersonal skills as a predictor of therapist success. J Clin Psychol.

[35] Gibbons JS, Butler J, Urwin P, Gibbons JL (1978). Evaluation of a social work service for self-poisoning patients. Br J Psychiatry.

[36] Hawton K, Catalan J (1981). Psychiatric management of attempted suicide patients. Br J Hosp Med.

[37] Townsend E, Hawton K, Altman DG, Arensman E, Gunnell D, Hazell P (2001). The efficacy of problem-solving treatments after deliberate self-harm: meta-analysis of randomized controlled trials with respect to depression, hopelessness and improvement in problems. Psychol Med.

[38] Suominen KH, Isometsa ET, Ostamo AI, Lonnqvist JK (2002). Health care contacts before and after attempted suicide. Soc Psychiatry Psychiatr Epidemiol.

[39] Runeson B (2001). Parasuicides without follow-up. Nord J Psychiatry.

[40] Cooper J, Murphy E, Jordan R, Mackway-Jones K (2008). Communication between secondary and primary care following self-harm: are National Institute of Clinical Excellence (NICE) guidelines being met?. Ann Gen Psychiatry.

[41] Gilbody S, House A, Owens D (1997). The early repetition of deliberate self harm. J R Coll Physicians Lond.

[42] Fleischmann A, Bertolote JM, Wasserman D, De LD, Bolhari J, Botega NJ (2008). Effectiveness of brief intervention and contact for suicide attempters: a randomized controlled trial in five countries. Bull World Health Organ.

