# Supplementary material for: Structured follow-up by general practitioners after deliberate self-poisoning: a randomised controlled trial
Source: BMC Psychiatry. 2015 Oct 14;15:245. doi: 10.1186/s12888-015-0635-2 (PMC4604741; doi:10.1186/s12888-015-0635-2)
Supplement: Additional file 1: — Guideline intervention. (PDF 79 kb) [file 12888_2015_635_MOESM1_ESM.pdf]

Guidelines for GPs in intervention group

Project guideline for general practitioners participating in the trial "Follow-up after self-poisoning: a randomised controlled clinical trial"

The intervention consists of regular consultations during a six-month period after the episode of self-poisoning that required hospitalisation: one week after discharge from hospital, each month for the next three months and finally two consultations six weeks apart. (This is a systematic additional component, and must not conflict with individually and needed contacts based on the physician's own assessments).

1) At the time of discharge, the hospital ward will arrange a consultation at the GP's practice and at the same time send a medical record. If this is not possible, a consultation will be aimed for within two weeks. If the patient does not attend, the GP should contact the patient by telephone and schedule a new appointment.

2) We ask you to clarify the following with the patient:

   - The reason for the episode
   - The main problem
   - Suicidal thoughts/ plans and possible control over these
   - Treatment
     - Is the patient involved in a treatment plan?
     - Is the treatment functioning?
     - If the treatment is not functioning, what is the cause?
     - Would it be useful to encourage the patients to follow treatment?
     - Should the person responsible for treatment be contacted to discuss the problem?

3) Does the patient need particular help now?

4) Schedule time for further consultations now (appointment or telephone contact)

5) Ensure that the patient can contact you if a new crisis emerges. Consider providing a contact number: if possible your own mobile number.

6) If you are in need of supervision or want to discuss problems, please contact nn at telephone no. n.
