# Supplementary material for: Structured follow-up by general practitioners after deliberate self-poisoning: a randomised controlled trial
Source: BMC Psychiatry. 2015 Oct 14;15:245. doi: 10.1186/s12888-015-0635-2 (PMC4604741; doi:10.1186/s12888-015-0635-2)
Supplement: Additional file 2: — Europep questionnaire. (PDF 38 kb) [file 12888_2015_635_MOESM2_ESM.pdf]

EUROPEP questionnaire

How do you perceive your general practitioner and the GPs office when it comes to..

(Scores: 1= very dissatisfied up to 5= very satisfied, 6= The question does not fit)

1. Making you feel you had time during consultations
2. Interest in your personal situation
3. Making it easy for you to tell him or her about your problems
4. Involving you in decisions about medical care
5. Listening to you
6. Keeping your records and data confidential
7. Quick relief of your symptoms
8. Helping you to feel well so that you can perform your normal daily activities
9.Thoroughness
10. Physical examination
11. Provide preventing measures (health check or vaccine)
12. Explaining the purpose of tests and treatments
13. Telling you what you wanted to know about your symptoms and/or illness
14. Help you to deal with emotional problems related to my health problems
15. Helping you understand the importance of following his or her advice
16. Knowing what s/he had done or told you during previous contacts
17. Prepare you for what to expect at specialist or hospital
18. The helpfulness of the staff (other than the doctor)
19. Getting an appointment to suit you
20. Get through on the telephone
21. Possibility to talk with the GP on telephone
22. Waiting time in waiting room
23. Providing quick services for urgent health problems

Scores item 24 and 25: 1= Totally disagree, 2= Rather disagree, 3= Neither agree or disagree, 4= Rather agree, 5= Totally agree 6= The question does not fit

24. Can recommend GP to friends
25. See no reason to change to other GP
